# Supplementary figures and images for: Neutral amino acid transporter SLC38A2 protects renal medulla from hyperosmolarity-induced ferroptosis
Source: eLife. 2023 Feb 1;12:e80647. doi: 10.7554/eLife.80647 (PMC9949798; doi:10.7554/eLife.80647)

**Control(Pex3)**

**p-SNAT2-C-EGFP**

SNAT2

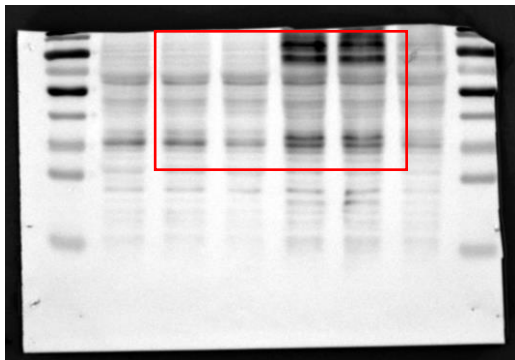

$\beta$ -actin

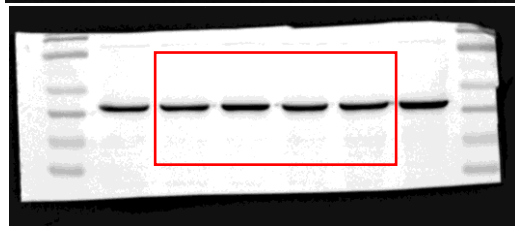

**Control(Pex3)**

**p-SNAT2-C-EGFP**

SNAT2

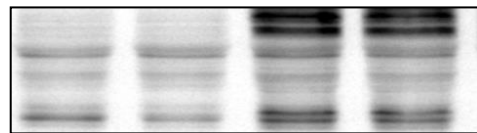

100 kDa

55 kDa

$\beta$ -actin

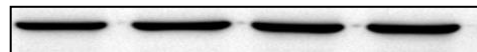

43 kDa

Supplement: Figure 2—figure supplement 4—source data 1. [file elife-80647-fig2-figsupp4-data1.zip › Figure 2-figure supplement 4-source data-1/Uncropped gels or blots/Figure 2-figure supplement 4B-uncropped gels or blots.pdf]

SNAT2 WT 禁水 24h 高通量 4

Con      WD

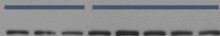

$\beta$ -actin

18-3-26

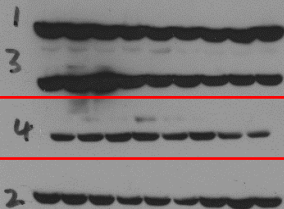

Medulla

Con

WD

SNAT2

56 kDa

$\beta$ -actin

43 kDa

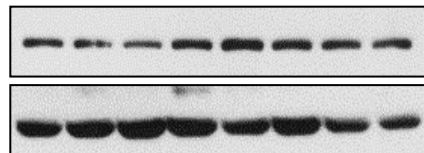

Supplement: Figure 3—source data 1. [file elife-80647-fig3-data1.zip › Figure 3-source data-1/Uncropped gels or blots/Figure 3C-uncropped gels or blots.pdf]

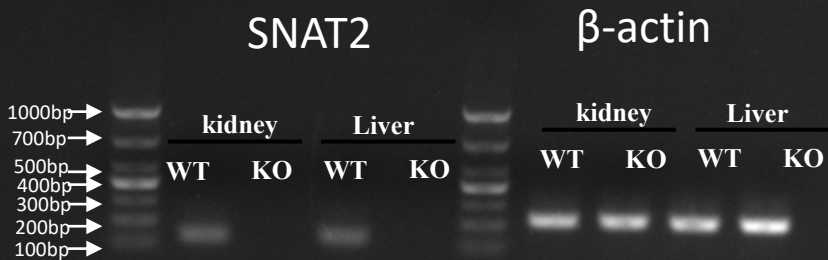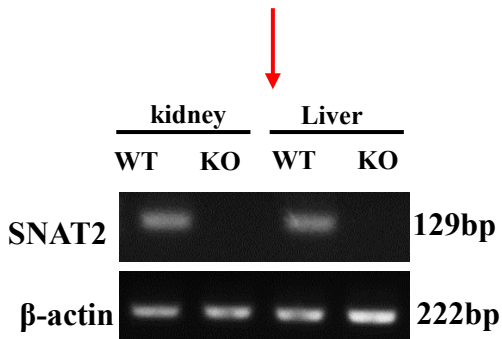

Supplement: Figure 4—source data 1. [file elife-80647-fig4-data1.zip › Figure 4-source data-1/Uncropped gels or blots/Figure 4B-uncropped gels or blots.pdf]

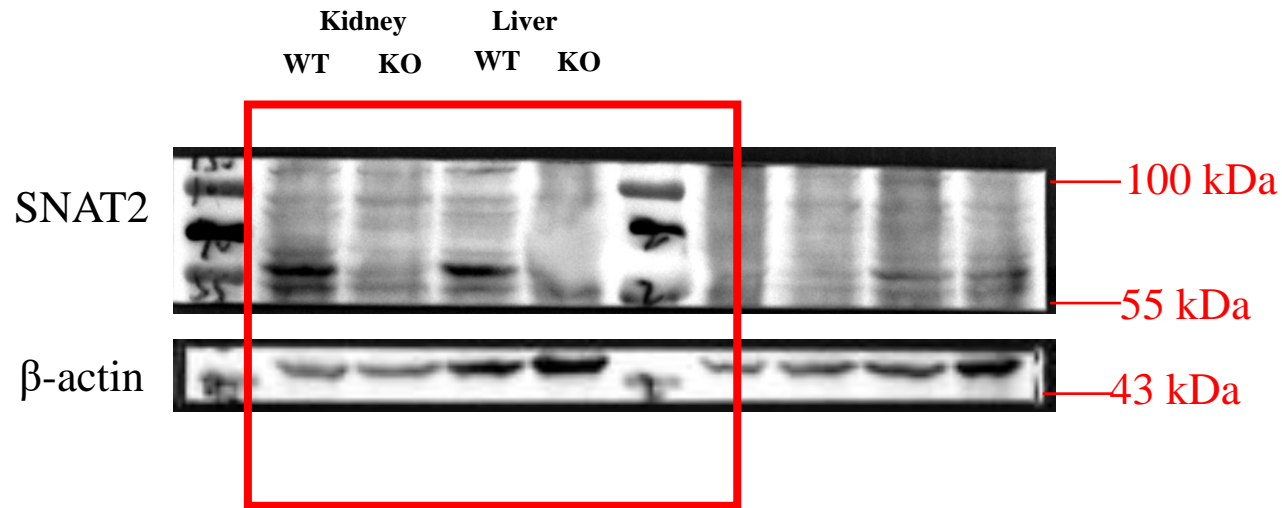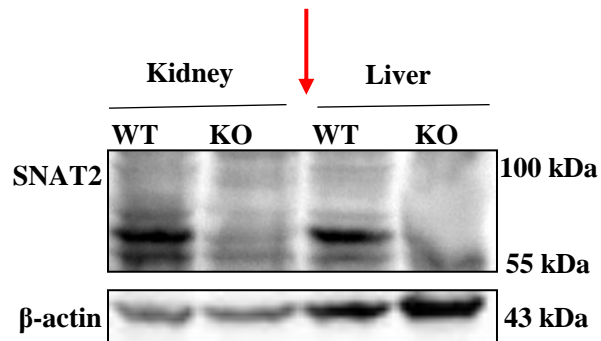

Supplement: Figure 4—source data 1. [file elife-80647-fig4-data1.zip › Figure 4-source data-1/Uncropped gels or blots/Figure 4C-uncropped gels or blots.pdf]

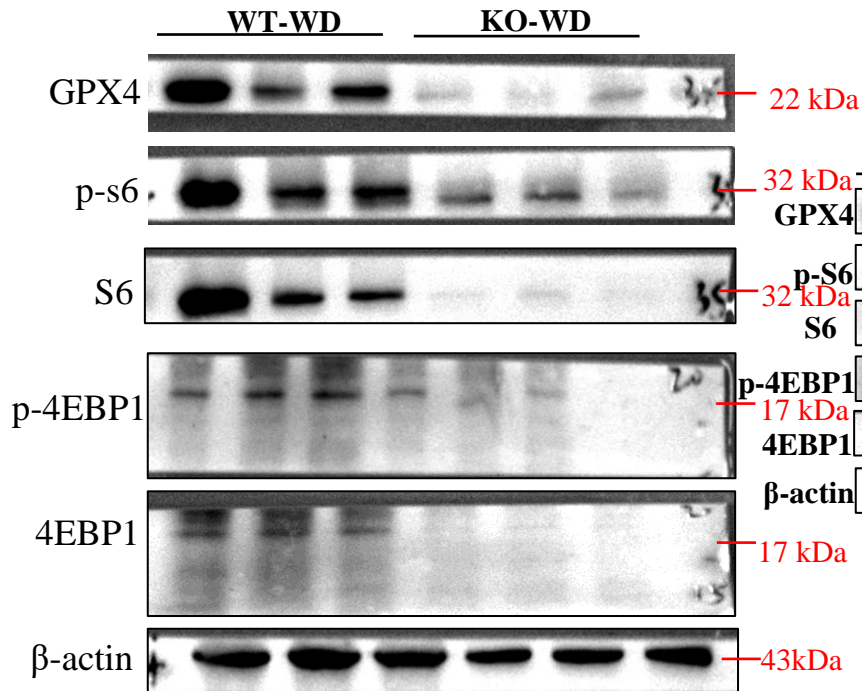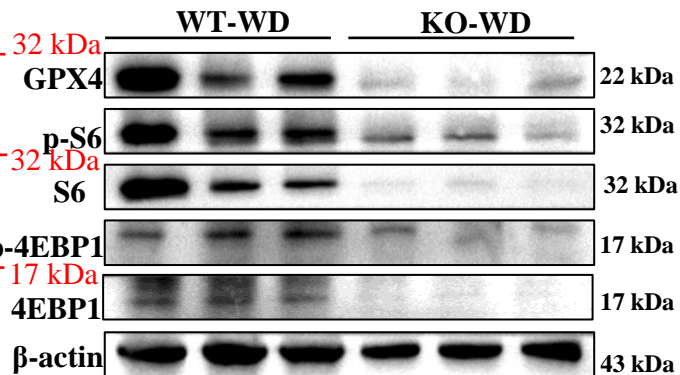

Supplement: Figure 4—source data 1. [file elife-80647-fig4-data1.zip › Figure 4-source data-1/Uncropped gels or blots/Figure 4L-uncropped gels or blots.pdf]

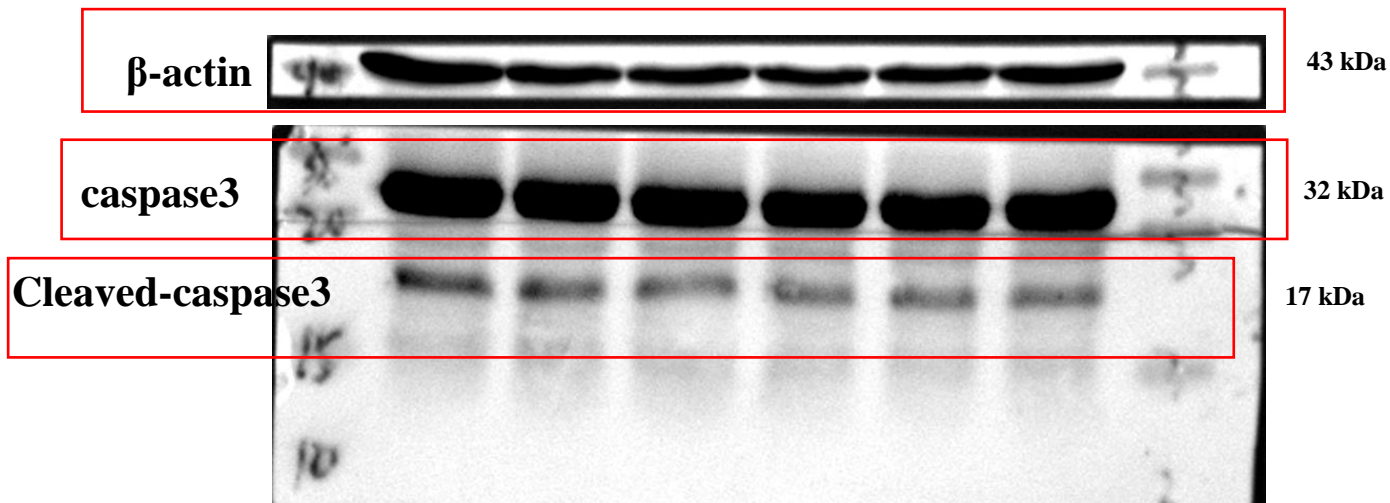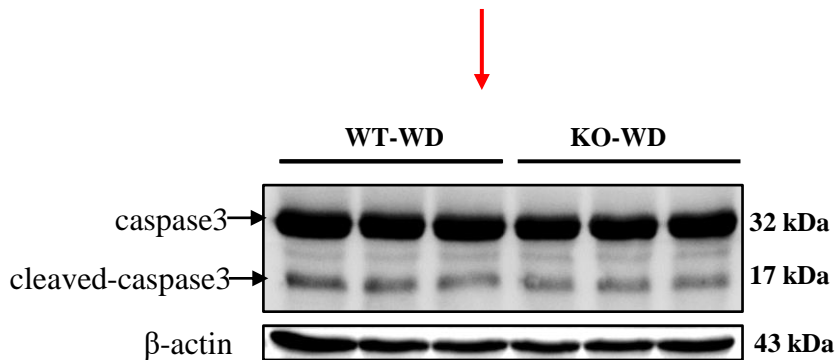

Supplement: Figure 4—figure supplement 3—source data 1. [file elife-80647-fig4-figsupp3-data1.zip › Figure 4-figure supplement 3-source data-1/Uncropped gels or blots/Figure 4-figure supplement 3B-uncropped gels or blots.pdf]

**SNAT2**

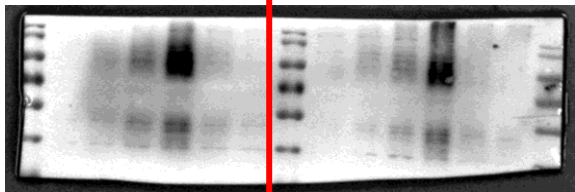

**$\beta$ -actin**

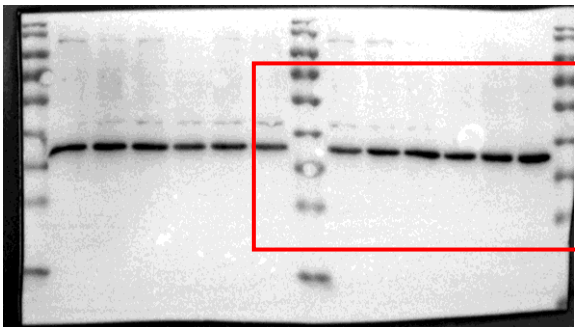

mOsm 300-400-500-600-700-800

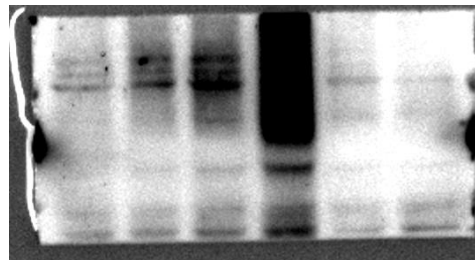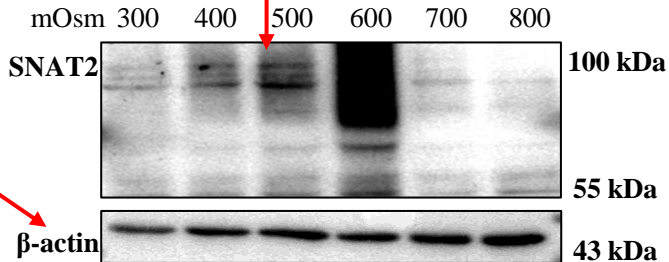

Supplement: Figure 5—source data 1. [file elife-80647-fig5-data1.zip › Figure 5-source data-1/Uncropped gels or blots/Figure 5B-uncropped gels or blots.pdf]

hours      0      3      6      9      12      24

SNAT2

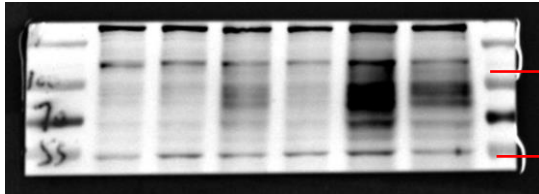

100kDa

55kDa

$\beta$ -actin

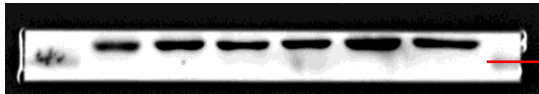

40kDa

hours

0      3      6      9      12      24

SNAT2

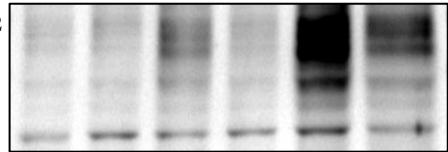

100 kDa

55 kDa

$\beta$ -actin

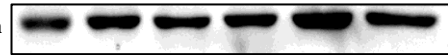

43 kDa

Supplement: Figure 5—source data 1. [file elife-80647-fig5-data1.zip › Figure 5-source data-1/Uncropped gels or blots/Figure 5F-uncropped gels or blots.pdf]

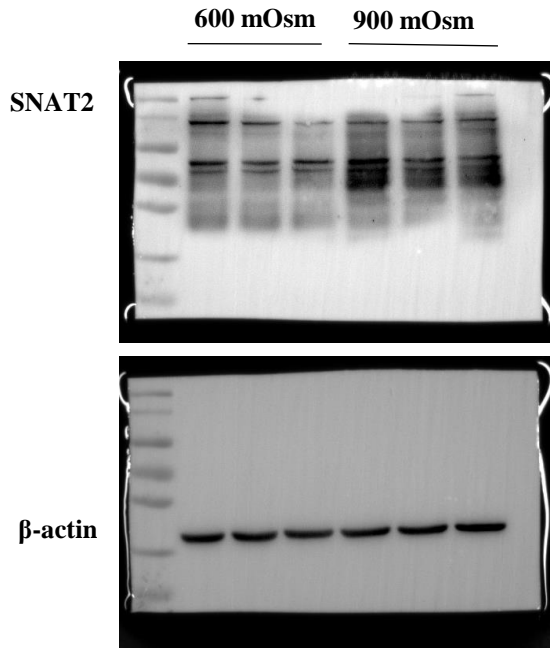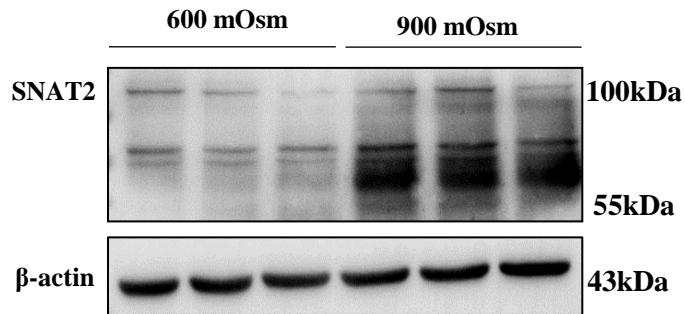

Supplement: Figure 5—figure supplement 1—source data 1. [file elife-80647-fig5-figsupp1-data1.zip › Figure 5-figure supplement 1-source data-1/Uncropped gels or blots/Figure 5-figure supplement 1D-uncropped gels or blots.pdf]

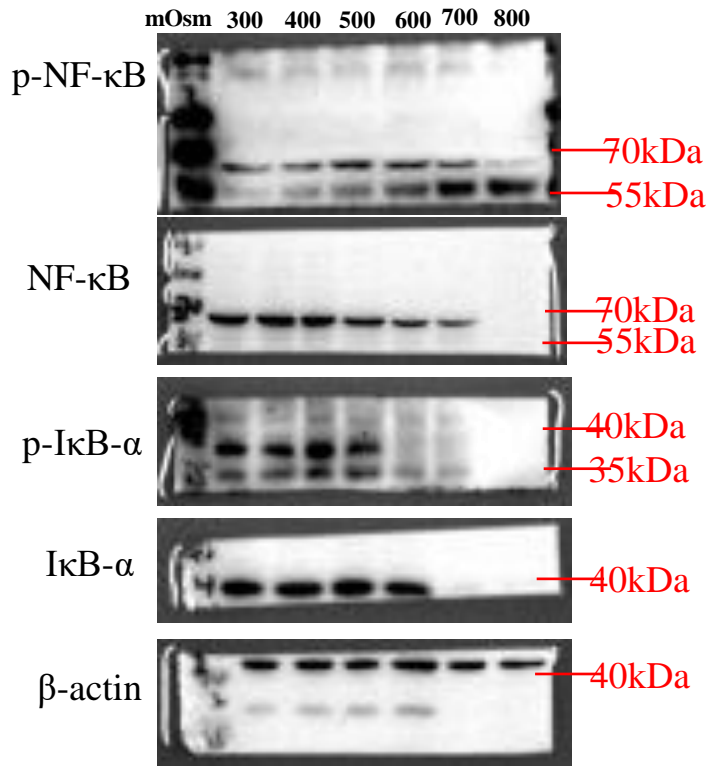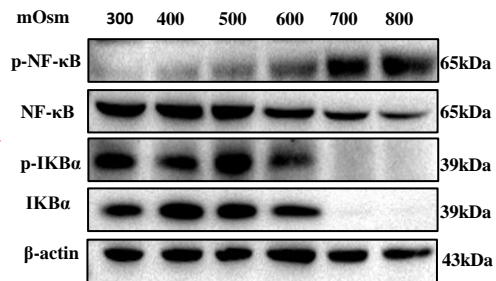

Supplement: Figure 6—source data 1. [file elife-80647-fig6-data1.zip › Figure 6-source data-1/Uncropped gels or blots/Figure 6A-uncropped gels or blots.pdf]

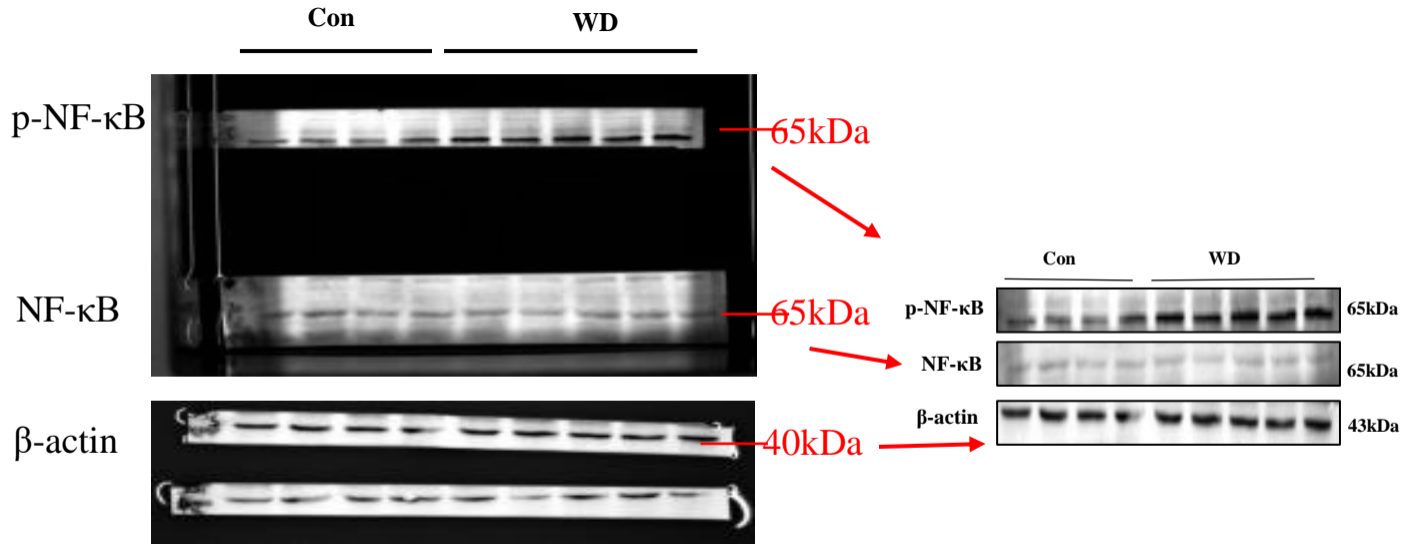

Supplement: Figure 6—source data 1. [file elife-80647-fig6-data1.zip › Figure 6-source data-1/Uncropped gels or blots/Figure 6C-uncropped gels or blots.pdf]

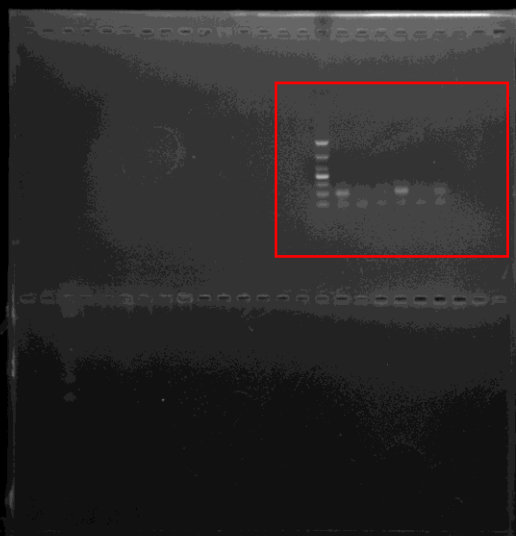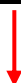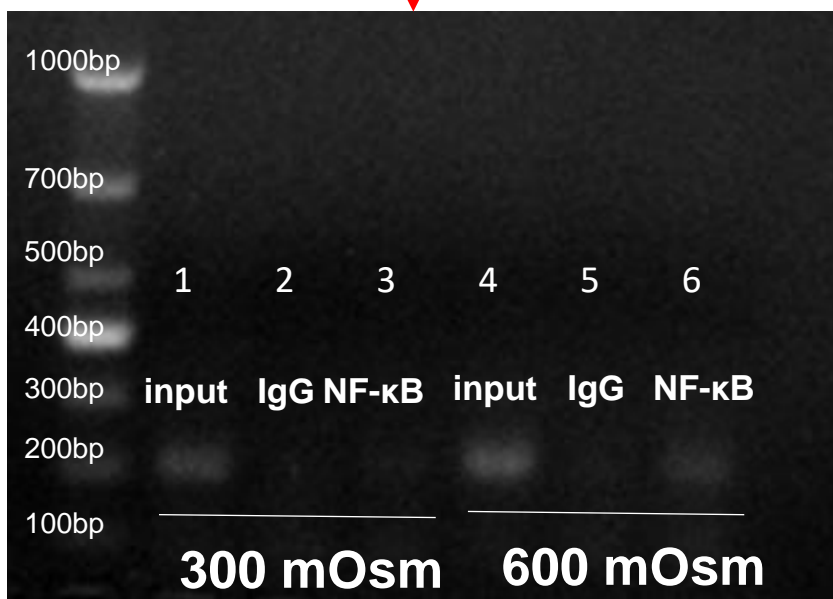

Supplement: Figure 6—source data 1. [file elife-80647-fig6-data1.zip › Figure 6-source data-1/Uncropped gels or blots/Figure 6F-uncropped gels or blots.pdf]

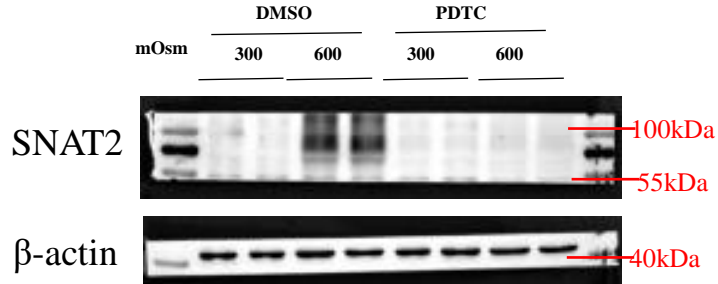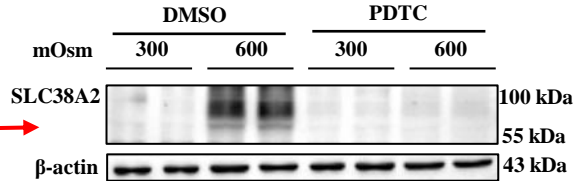

Supplement: Figure 6—source data 1. [file elife-80647-fig6-data1.zip › Figure 6-source data-1/Uncropped gels or blots/Figure 6I-uncropped gels or blots.pdf]

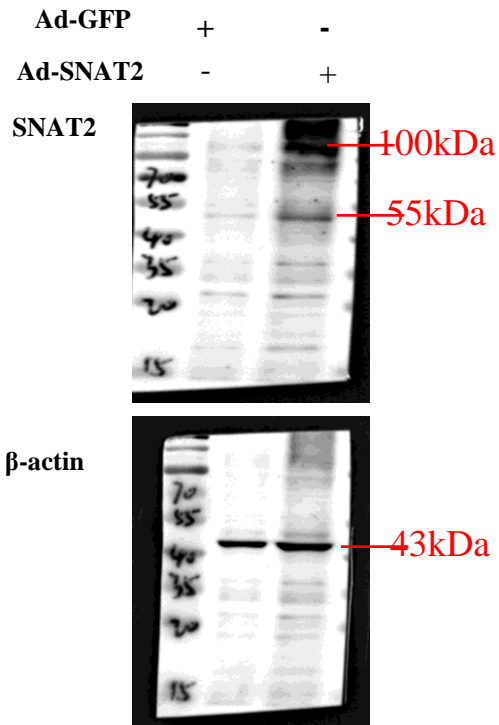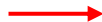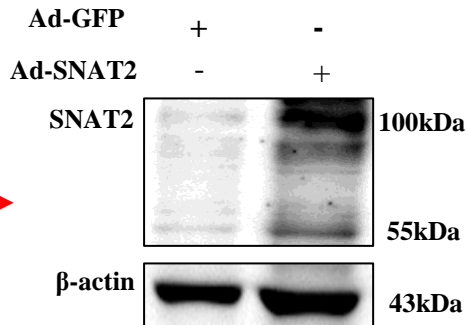

Supplement: Figure 7—source data 1. [file elife-80647-fig7-data1.zip › Figure 7-source data-1/Uncropped gels or blots/Figure 7B-uncropped gels or blots.pdf]

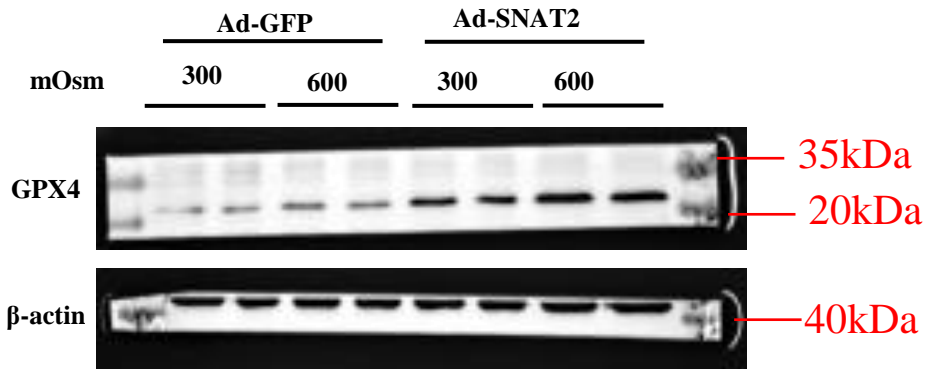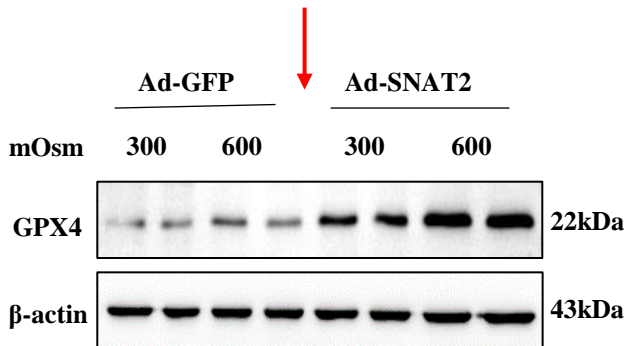

Supplement: Figure 7—source data 1. [file elife-80647-fig7-data1.zip › Figure 7-source data-1/Uncropped gels or blots/Figure 7E-uncropped gels or blots.pdf]

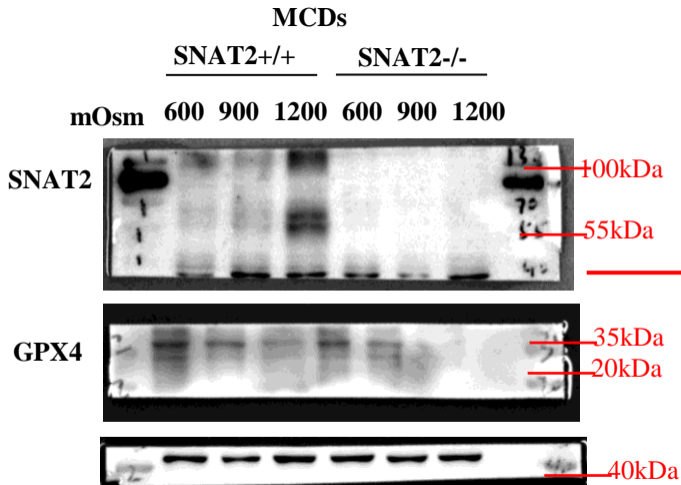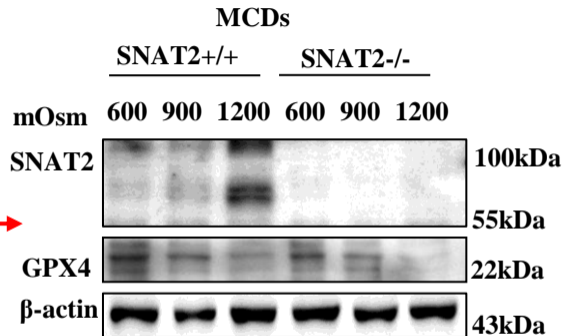

Supplement: Figure 8—source data 1. [file elife-80647-fig8-data1.zip › Figure 8-source data-1/Uncropped gels or blots/Figure 8E-uncropped gels or blots.pdf]

siNC siRNA-SNAT2

SNAT2

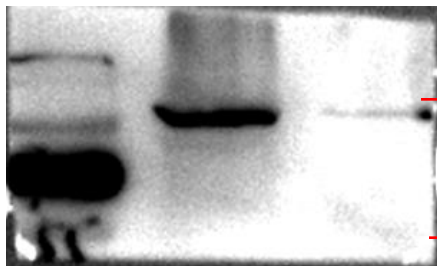

100 kDa

55 kDa

$\beta$ -actin

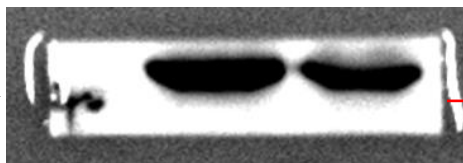

40 kDa

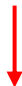

siNC siRNA-SNAT2

SNAT2

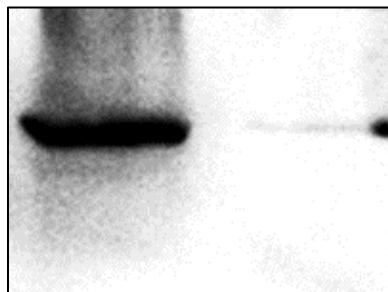

100 kDa

56 kDa

$\beta$ -actin

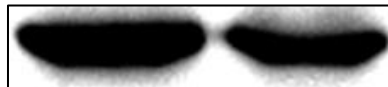

43 kDa

Supplement: Figure 8—figure supplement 1—source data 1. [file elife-80647-fig8-figsupp1-data1.zip › Figure 8-figure supplement 1-source data-1/Uncropped gels or blots/Figure 8-figure supplement 1B-uncropped gels or blots.pdf]

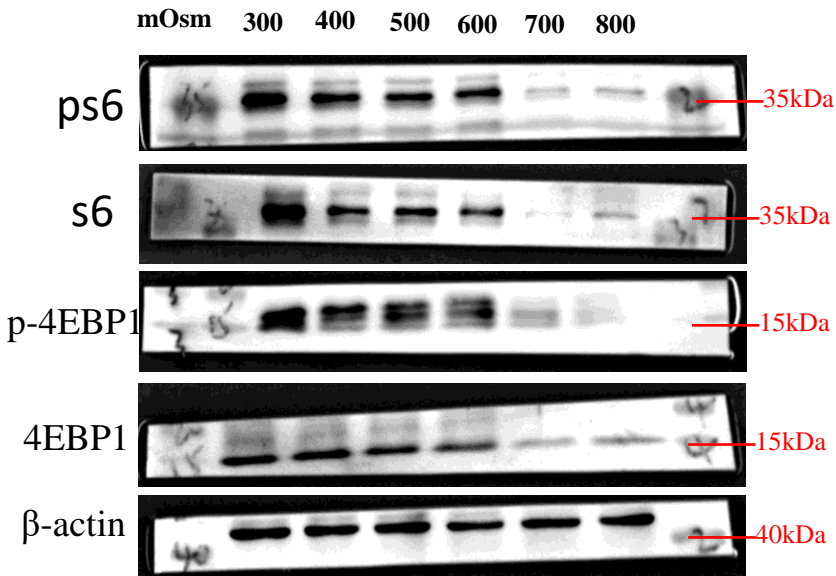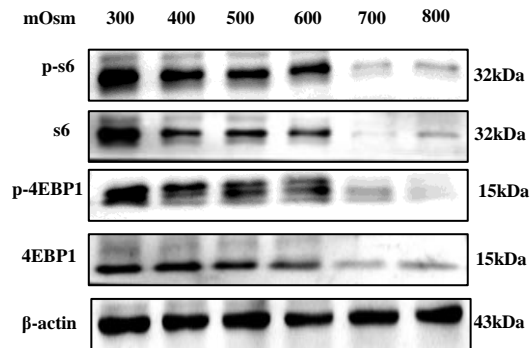

Supplement: Figure 9—source data 1. [file elife-80647-fig9-data1.zip › Figure 9-source data-1/Uncropped gels or blots/Figure 9A-uncropped gels or blots.pdf]

| mOsm     | 300 |   | 600 |   |
|----------|-----|---|-----|---|
| Ad-GFP   | +   | - | +   | - |
| Ad-SNAT2 | -   | + | -   | + |

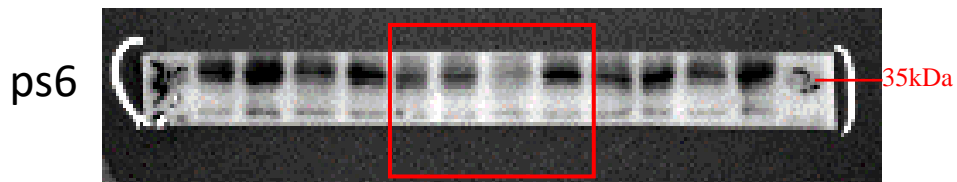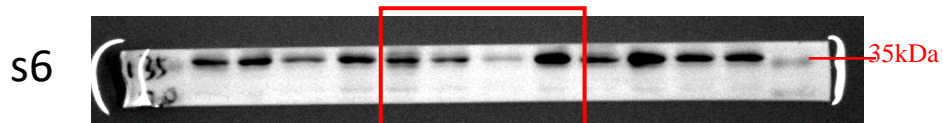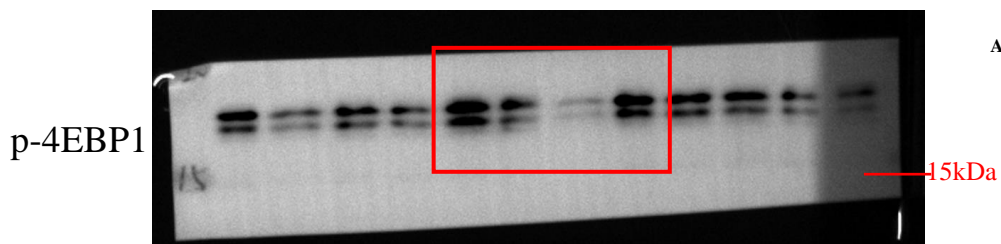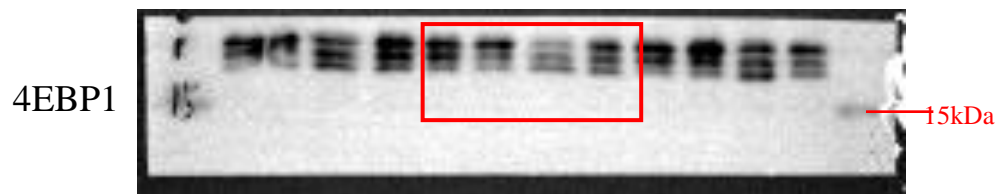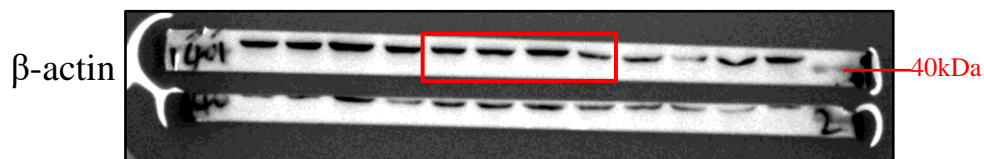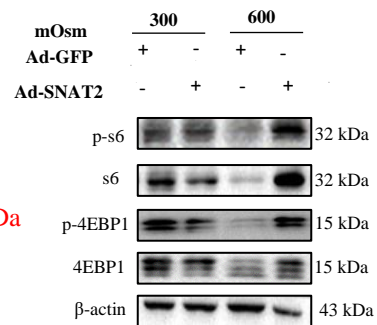

Supplement: Figure 9—source data 1. [file elife-80647-fig9-data1.zip › Figure 9-source data-1/Uncropped gels or blots/Figure 9F-uncropped gels or blots.pdf]

| mOsm | DMSO |     | Torin1 |     | Rapamycin |     |
|------|------|-----|--------|-----|-----------|-----|
|      | 300  | 600 | 300    | 600 | 300       | 600 |

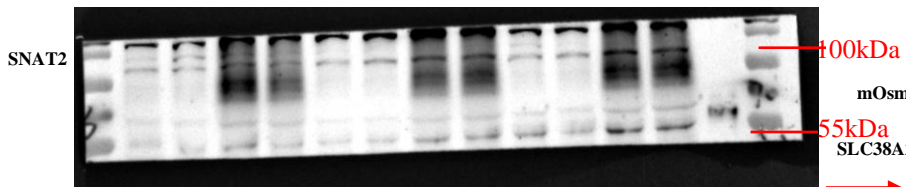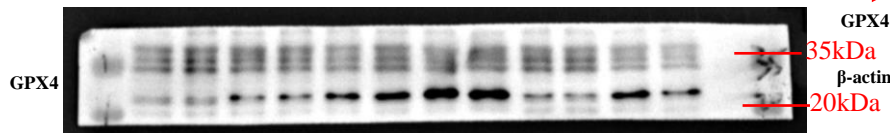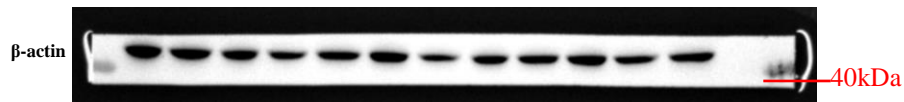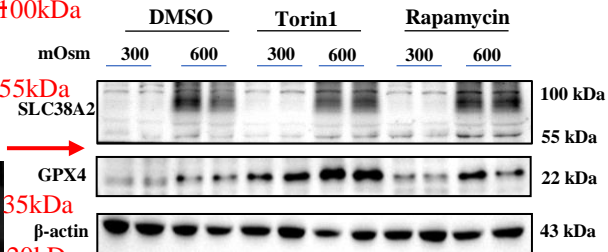

Supplement: Figure 9—figure supplement 1—source data 1. [file elife-80647-fig9-figsupp1-data1.zip › Figure 9-figure supplement 1-source data-1/Uncropped gels or blots/Figure 9-figure supplement 1B-uncroped gels or blots.pdf]
